# Efficacy of a training programme to support the application of the g*uideline evidence-based health information*: study protocol of a randomised controlled trial

**DOI:** 10.1186/s13063-020-04287-1

**Published:** 2020-05-25

**Authors:** Julia Lühnen, Birte Berger-Höger, Burkhard Haastert, Jana Hinneburg, Jürgen Kasper, Anke Steckelberg

**Affiliations:** 1 Institute for Health and Nursing Science, Martin Luther University Halle-Wittenberg, Magdeburger Str. 8, 06112 Halle (Saale), Germany; 2 mediStatistica, Lambertusweg 1b, 58809 Neuenrade, Germany; 3 Faculty of Health Sciences, Department of Health and Caring Sciences, University of Tromsø, Postbox 6050, Langnes, Norway; 4 Department of Nursing and Health Promotion, OsloMet – Oslo Metropolitan University, Oslo, Norway

**Keywords:** Health information, Guideline implementation, training programme, Evidence-based medicine, Guideline evidence-based health information

## Abstract

**Background:**

The evidence-based guideline entitled *guideline evidence-based health information* emerged from the *German Network for Evidence-based Medicine (DNEbM)* and was published in February 2017. The guideline addresses providers of health information and its goal is to improve the quality of health information. In addition, we explored the competences of providers of health information and developed a training programme.

The aim of this study is to evaluate the efficacy of a training programme addressing providers of health information to support the application of the *guideline evidence-based health information*. We expected the intervention to improve the quality of health information in comparison to the provision of the guideline on its own.

**Methods/design:**

The trial uses a superiority randomised control group design with 10 months’ follow-up. Twenty-six providers of health information (groups with up to ten members) will be enrolled to compare the intervention (guideline and training programme) with usual care (a publicly available guideline). The 5-day training programme comprises an evidence-based medicine training module and a module to prepare the application of the guideline. The primary outcome parameter is the quality of the health information. Quality is operationalised as the extent of adherence to the guideline’s recommendations. Each provider will prepare a single health information item informing a health-related decision on a topic freely chosen before randomisation. The quality of this information will be rated using the *Mapping Health Information Quality* (MAPPinfo) Checklist. An accompanying process evaluation will then be conducted.

**Discussion:**

The study results should show whether the efficacy of the intervention justifies implementation of the training programme to enhance health information developers’ competences in evidence-based medicine and to ensure high-quality evidence-based health information (EBHI) in the long term.

**Trial registration:**

ISRCTN registry, ID: ISRCTN96941060. Registered on 7 March 2019.

## Background

In 2017, the *German Network for Evidence-based Medicine* (Deutsches Netzwerk Evidenzbasierte Medizin (DNEbM) *e.V.*) published an evidence-based guideline entitled *guideline evidence-based health information* [1]. The guideline addresses health information providers, regardless of the indications or target groups that are focussed on. The guideline aims to improve the quality of health information. Evidence-based health information (EBHI) is the prerequisite for informed decision-making. An informed decision is based on relevant knowledge and is consistent with the patient’s values and preferences [2]. An *informed choice* should be defined as a patient-relevant outcome measure of shared decision-making processes [3]. Most people prefer being involved in decision-making processes in healthcare and want to receive more information [4]. In addition to the ethical rights [5], the German act on patients’ rights [6] requires the provision of understandable and comprehensive information before giving informed consent to any therapeutic, diagnostic or screening interventions. The criteria for EBHI have been comprehensively described [7, 8] but the implementation is still insufficient [9, 10]. In 2016, the DNEbM, section *patient information and participation,* released the second edition of the *good practice guidelines for health information *(GPGI), which suggest the necessity of standards for EBHI [11]. The intervention for implementing the *guideline evidence-based health information* will also refer to the GPGI*.*

The development of the evidence-based *guideline evidence-based health information* was carried out according to manuals and handbooks on guideline development [12–14], approaches to reform the guideline development processes in order to reduce bias [15–21], and also according to GRADE (*Grading of Recommendations, Assessment, Development and Evaluation*) [22]. The guideline development group consisted of providers of health information, health scientists and patient representatives. Based on GRADE [23], the guideline development group defined key questions related to content, presentation and the development process of health information. Systematic literature searches, quality assessments and data analyses were performed in the *Unit of Health Science and Education* of the University of Hamburg. GRADE evidence profiles [24] were drawn up and proposals for the wording of the recommendations were made. The guideline development group discussed and established consensus about the recommendations and additional texts in the guideline. The guideline comprises general and ethical requirements, which were approved by the guideline development group as obligate aspects of EBHI, as well as recommendations based on the systematic evidence syntheses. The general and ethical requirements include the development process, target group orientation and the content of EBHI*.* The 21 recommendations mainly address how the information is presented, such as the presentation of frequencies or the use of pictures and graphics. The first version of the guideline and the methodological report were released for public consultation in autumn 2016. After revision and final consent of the guideline development group, the current version was published [1].

In general, guidelines may help to establish quality criteria and to improve practice [25]. Various strategies regarding implementation have been discussed and it would seem that implementation in combination with a training programme might be successful [26]. Especially for the implementation of the *guideline evidence-based health information,* training the providers seems to be an appropriate method. Interviews with providers revealed shortcomings regarding their competences in evidence-based medicine (ebm) [27]. However, these competences are prerequisites for fulfilling the requirements for the development and presentation of EBHI. Therefore, we have developed a training programme for health information providers. The programme comprises two modules: an ebm training module and a module to prepare the application of the guideline. We have developed a blended-learning programme considering existing face-to-face training programmes and tested it for acceptance and feasibility with providers of health information in a qualitative pilot study [28].

## Objectives

The aim of this study is to evaluate the efficacy of a training programme addressing providers of health information to support the application of the *guideline evidence-based health information*. The observation unit is the provider. We expect the intervention to improve the quality of health information in comparison to the provision of the guideline on its own.

## Methods/design

The reporting of this protocol follows the criteria of the Standard Protocol Items: Recommendations for Interventional trials (SPIRIT) [29] and the UK Medical Research Council (MRC) framework for the development and evaluation of complex interventions [30]. For the completed SPIRIT Checklist, see Additional file 1.

### Design

The trial uses a superiority randomised control group design, comparing two groups of equal sizes. In addition, the trial includes a formative evaluation seeking to fully understand the involved mediating mechanisms as well as the barriers towards the implementation process.

### Setting and participants

The intervention is made available on-site at providers of publicly available health information (e.g. health insurance companies, self-help associations or foundations, health portals, hospitals, rehabilitation clinics, nursing facilities and physicians) in German-speaking areas, mainly in Germany and Austria. Providers are defined as institutions or working groups rather than individuals and may, therefore, comprise several individuals (e.g. up to ten).

### Eligibility and recruitment

#### Eligibility criteria

Providers will be eligible if they are responsible for the production and publishing of health information. The use of external services (e.g. counselling by experts, graphic design, external performing of pilot tests or external literature searches) is not a reason for exclusion, as long as the provider is designated as the responsible publisher/editor. Providers should have published information both regularly and currently. Therefore, they are eligible if they have published any information in the last 18 months. In addition, at least a single health information item produced in the last 3 years has to fulfil the following criteria: The information:

- has to inform a health-related decision
- has to address patients or medical laypersons
- has to discuss different options regarding one specified health problem
- does not inform about a single option, procedure or healthcare system and
- it does not give general advice on health and wellbeing

It is essential that the participating providers agree to comply with the training programme and the production of one item of health information. Above all, the providers will be notified about the purpose and schedule of the intervention and their obligation to produce the new health information within 10 months.

Members of the working groups on the *guideline evidence-based health information* or the GPGI are not eligible. The same applies to providers producing exclusively (drug)-fact(s) boxes or offering counselling services such as medical online consulting websites, health related blogs, forums or communities or (online) encyclopaedias.

Individuals in the institution or working group who are closely involved in the development of the health information will be included in the study and, if possible, all of them should attend the training. Staff turnover or other changes during the study period will be documented. New staff members will also have the opportunity to attend the training. The providers themselves will choose the participants according to their working processes and resources. No further eligibility criteria will be defined.

#### Recruitment

**Fig. 1:**
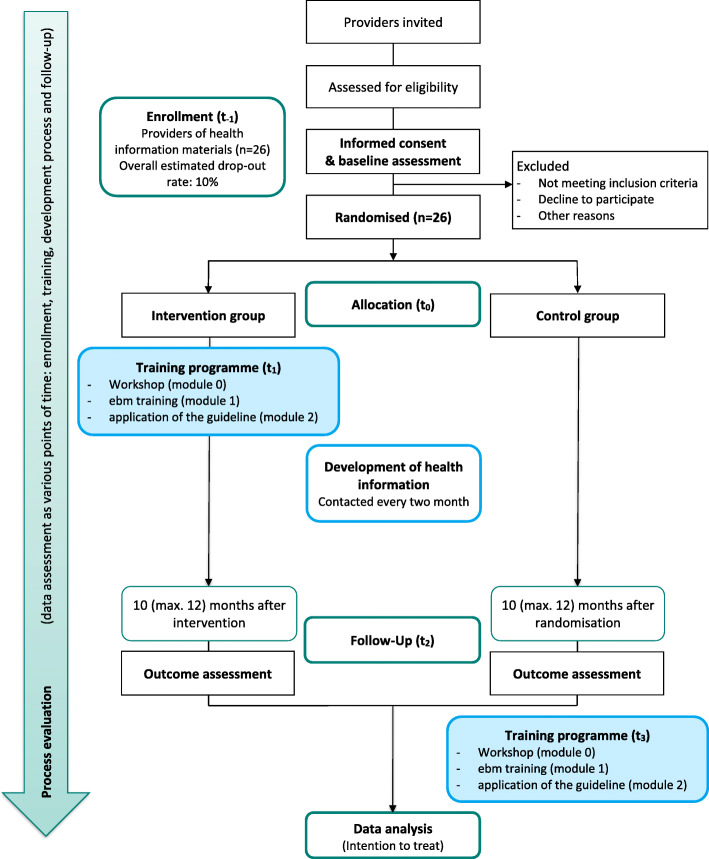
Study flow

Providers will be identified by means of Internet searches. We assume that most providers have a website, even if they issue printed material. We will search via *Google* and *MetaGer* with different terms for online health information and webpages that refer to information (print or pdf). The providers’ data will be collected via the information and webpages (e.g. contact details). In addition, data of well-known providers will be listed. The identified providers will be assessed for their eligibility (membership in the working groups *guideline evidence-based health information* and/or GPGI, the amount and kind of information published by them in the last 3 years). Contact details and the persons in charge will be identified online or by telephone. We will continue the search until 100 eligible providers are identified. These will receive a cover letter informing them briefly about the study and the *guideline evidence-based health information* and including an invitation to take part in this project (see study flow, Fig. 1). Providers who do not reply within 2 weeks will be contacted personally by telephone with an offer of further information. If providers are interested, a telephone or online meeting will be scheduled to assess further eligibility criteria and to give an overview of the course of the study.

Afterwards, a personal meeting will be set up with eligible providers who intend to participate. The meeting is to give further information, to obtain informed consent, to assess baseline data and to perform the allocation. Further information about the study will be presented with special reference to the relevance of EBHI, the intervention and the primary outcome. The providers have to know about the requirements and advantages associated with their participation. Requirements are, for example: releasing employees from work for the training programme, providing time and financial resources to develop a single health information item in the study period, possibly with more effort in the intervention group (IG) in order to realise the guideline recommendations. In return, a final symposium with the presentation of the best information will be scheduled after completion of the project. With their consent, the providers’ logos will be published on the project webpage after completing the study. In addition, the health information produced in the study may be published on the webpage along with the results of the quality rating. After receiving notification about requirements and incentives, the providers must agree to comply with the training programme and to produce within 10 months one item of health information that informs about a health-related decision. In addition, written informed consent will be obtained from the individual participants and baseline data will be assessed by the researchers. Recruitment and randomisation will be performed consecutively.

### Randomisation and blinding

The concealed allocation of participating provider groups to either the intervention or the control group (CG) will be determined by randomisation, using a computer-generated list with randomly permuted blocks of length 2, 4, 6 or 8. An independent external person will prepare sealed, opaque envelopes. After the baseline assessment of the respective provider group, the researchers will open the sealed, opaque envelope and reveal the centre’s allocation on-site.

Due to the nature of the intervention, blinding of the participating providers and the researchers conducting the training programme is not possible. However, the assessment of the primary endpoint and all analyses will be blinded towards group allocation.

### Interventions

#### Intervention group (IG)

The providers in the IG receive a training, which is intended to facilitate the appropriate use of the *guideline evidence-based health information*.

##### Guideline evidence-based health information

The guideline defines the quality criteria of EBHI. It comprises general and ethical requirements regarding the development process, target group orientation and content of EBHI as well as 21 evidence-based recommendations assigned to the topics: *presentation of frequencies*, *application of graphics*, *pictures and drawings*, *narratives*, *value clarification tools*, *formats* and *involvement of the target group*. Table 1 gives an overview of the guideline and its recommendations.

**Table 1:** Structure and content of the *guideline evidence-based health information*

Chapters	Content
1. The guideline project	Information on the *guideline evidence-based health information* and the guideline development process (e.g. aim and field of application, methods, up-dating procedures)
2. Development of EBHI	Description of the development process of evidence-based health information (EBHI) (2.1) and quality criteria regarding content and presentation of EBHI (2.2)
2.1 Development and evaluation of EBHI	Explanation of the development process of EBHI based on the four phases of the UK General Medical Council (UK MRC) framework for the development and evaluation of complex interventions [30]. *Development* and *piloting* are valued as mandatory for developing EBHI and *evaluation* and *implementation* as desirable
2.2. Quality criteria	21 recommendations based on evidence syntheses and a formal consensus processes
2.2.1 Target group orientation	Quality criteria regarding understandable language, accessibility of information, cultural differences and issues related to age and gender of the users, due to ethical requirements
2.2.2 Content requirements	The criteria on content and transparency were derived from the ethical guideline of the UK GMC, defining which information patients should receive before they consent to medical interventions [5]. In Germany, the requirements are legally anchored in the German Act on Patients’ Rights [6]
2.2.3 Presentation of frequencies	Recommendations (*n* = 5) for verbal and numerical presentation of frequencies
2.2.4 Application of graphics	Recommendations (*n* = 6) for the application and design of different types of graphics
2.2.5 Application of pictures	Recommendations (*n* = 5) for the application of anatomical pictures, cartoons, photos, pictographs and drawings
2.2.6 Application of narratives	Recommendation (*n* = 1) for the application of narratives
2.2.7 Application of value clarification tools	Recommendation (*n* = 1) for the application of value clarification tools
2.2.8 Formats	Recommendations (*n* = 2) for the application of interactive tools and fact boxes
2.2.9 Involvement of the target group	Recommendation (*n* = 1) for the involvement of the target group in the development process of EBHI

##### Workshop for persons in charge of providing health information (module 0)

Even though the actual target group of the training programme is people directly involved in developing and producing health information, our intervention also needs to address the providers on the level of responsible decision-makers and leaders in the organisation. Attitudes of these persons can be both facilitators and/or barriers to the implementation of the EBHI criteria. Therefore, they will be invited to a workshop with the purpose of discussing and reflecting on the benefits of using the EBHI criteria. In particular, the systemic structures required for developing EBHI will be addressed. If possible, the workshop (up to 2 h) should take place on-site directly after randomisation into the intervention group. If this is not possible, a second personal or online meeting will be scheduled.

##### Training programme (modules 1 and 2)

The free training programme is based on a problem-based instructional design that is learner-centred and enables learners to link theory with practice and to apply knowledge and skills to develop EBHI for a defined health problem for special target groups [31]. Case-based learning links theory to practice through the application of knowledge about the cases and by using inquiry-based learning methods [32]. We set up a case example about smoking cessation. This was chosen since it has relevance for several health professions and smoking is still a widely discussed topic. Moreover, (passive) smoking concerns everyone in daily life. Good health information is needed because most people in Germany try to quit smoking on their own, which is the less promising option [33]. People need to be informed about evidence-based methods for smoking cessation, such as counselling, medications (e.g. antidepressants) and nicotine replacement therapy. Therefore, it is also a relevant topic for providers of health information.

**Fig. 2:**
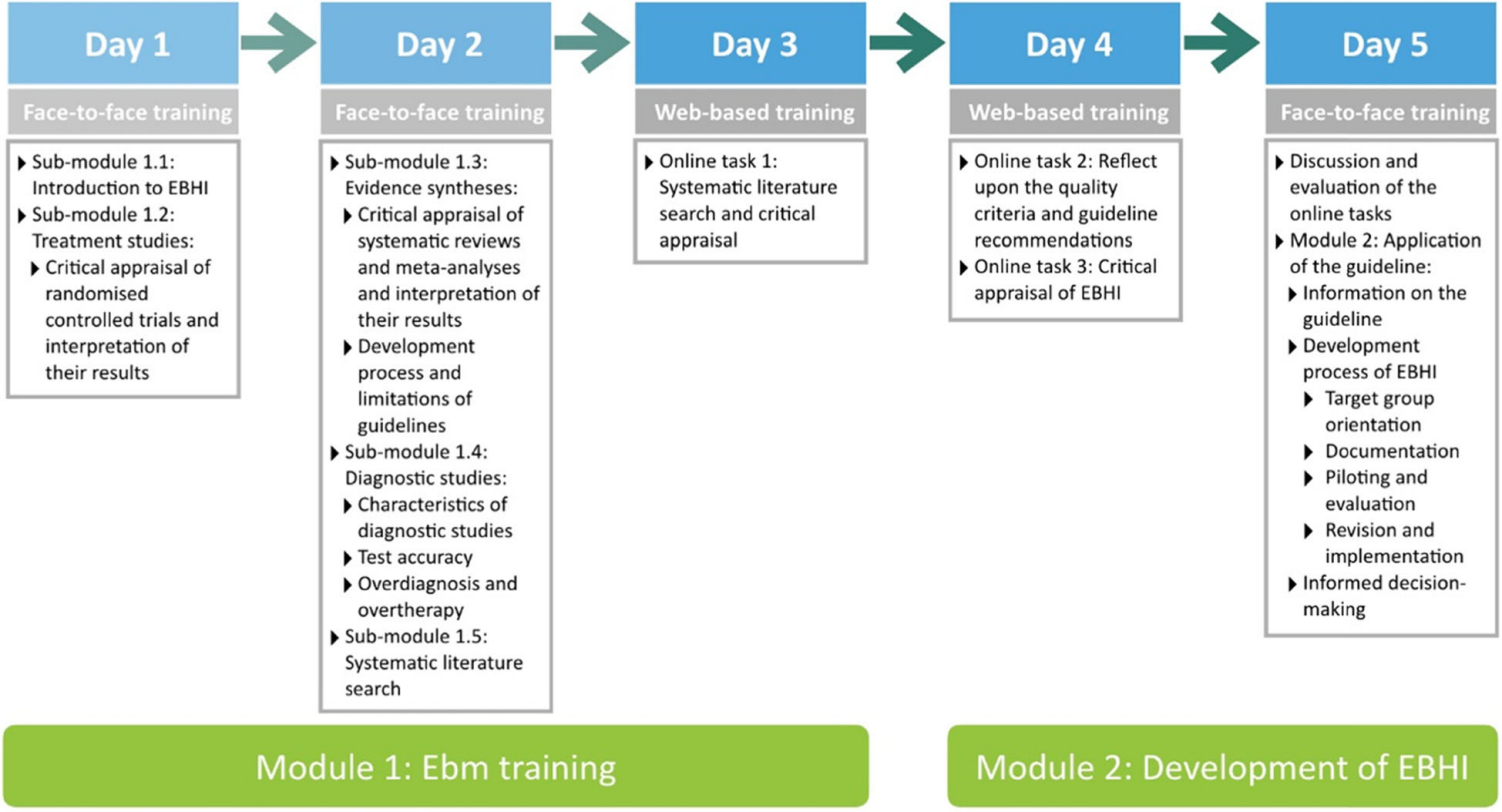
Modular training structure

The training programme comprises module 1 ‘ebm training’ and module 2 ‘application of the guideline’. It is designed in a blended-learning format [34] where face-to-face and web-based learning activities alternate (see Fig. 2).

The ebm training module is divided into five sub-modules: introduction, treatment studies, evidence syntheses and guidelines, diagnostic studies and systematic literature search. The teaching goals are broadly defined (Table 2).

**Table 2:** Teaching goals ‘ebm training’ and ‘application of the guideline’

**Module 1: ebm training**	
Sub-module	Goals
1.1 Introduction	• Participants gain an overview of the development process of evidence-based health information (EBHI) and reflect on their own practice
	• Participants start to consider EBHI as the prerequisite for informed decision-making
1.2 Treatment studies	• Participants understand the difference between association and causality and that randomised controlled trials (RCTs) are designed to establish a causal relationship
	• Participants know the characteristics of RCTs
	• Participants are able to interpret the results of RCTs and critically appraise them
1.3 Evidence syntheses and guidelines	• Participants are able to interpret the results and critically appraise systematic reviews and meta-analyses
	• Participants describe the development process of guidelines and are aware of their limitations
1.4 Diagnostic studies	• Participants are able to identify the major study designs for diagnostic studies
	• Participants are able to calculate and interpret test accuracy
	• Participants recognise the problem of over-diagnosis and over-treatment
1.5 Systematic literature search	• Participants are able to conduct systematic literature searches to identify appropriate literature matching their research question
**Module 2: application of the guideline**	
	• Participants are able to develop EBHI and document the development process
	• Participants know about and apply strategies for piloting EBHI
	• Participants consider EBHI as the prerequisite for informed decision-making

The *first module* (2 days of face-to-face training followed by 1 day of web-based training) aims to impart competences in searching for, selecting, critically appraising and extracting relevant literature according to the principles of ebm.

The *second module* is designed as an inverted classroom scenario (1 day of web-based training followed by one day of face-to-face training) [35]. In preparation for the second module, participants become acquainted with the guideline recommendations for the development of EBHI. Guideline recommendations are presented in an audio-visual form with illustrative examples. Participants can then intensify and improve their understanding of the criteria for EBHI by critically appraising an existing health information.

During the second face-to-face training, online tasks are discussed and further group exercises are undertaken in order to improve the understanding of the development and pilot testing of EBHI.

The participants are invited to reflect their current practice concerning the development of health information, taking into consideration the methods of evidence-based medicine and the guideline recommendations for EBHI.

For the *web-based learning scenario*, a learning management system (ILIAS) is used to provide web-based content. Features of the learning management system are slideshows and text sources combined with online tasks and (video) tutorials. Further information on the course content is provided. Participants are encouraged to upload their results and receive feedback during the face-to-face training. The group size for training should not exceed 20 participants. The training programme was pilot tested and revised according to the pilot study results [28].

#### Control group (CG)

Providers in the CG receive an optimised standard care in the form of a link and reference to the guideline evidence-based health information. They do not get access to the training programme or materials exceeding the guideline itself. They are offered the possibility of participating in the training programme after data collection is completed.

### Baseline data

#### Baseline characteristics

The baseline characteristics of the health information providers will be assessed on both the institutional and individual level. On the institutional level, the size of the organisation (number of employees in total and number of employees developing health information), number of health information publications in the last 3 years, main target group(s) and topic(s) of health information, funding of health information and the management of conflict of interests will be assessed. The responsible contact has to fill in a standardised questionnaire. On the level of individual participants, their sex, age, education status and English language skills will be assessed, along with their qualifications for the development of health information, the duration of their current position, and previous experiences and attitudes regarding ebm and EBHI. These participants also have to fill in a standardised questionnaire. In addition, a declaration about conflict of interests is required.

#### Baseline variables

##### Quality of health information

Each provider’s most recent health information, which meets the criteria of materials giving information towards a health-related decision, will be rated regarding quality using the *Mapping Health Information Quality* (MAPPinfo) Checklist.

##### Critical health literacy

Critical health literacy will be assessed using the *Critical Health Competence Test* (CHC test) [36]. The test is based on a 4 × 4 facets design. The first facet consists of four different and relevant content areas of healthcare. The second facet is built up with four subareas of competence, representing the underpinning theoretical structure of the critical health literacy construct. These categories are: A. Understanding medical concepts; B. Skills of searching literature; C. Basic statistics; and D. Design of experiments and sampling. Reliability of the test for the Rasch model (analysis of variance (ANOVA)) was 0.91 and for the single scenarios 0.71 (scenario 1); 0.78 (scenario 2); 0.75 (scenario 3) and 0.80 (scenario 4).

The CHC test comprises four different scenarios which allows testing at different time points using different scenarios. The test will be carried out at baseline and as part of the process evaluation after the training programme in the IG and the CG. Critical health literacy will be aggregated at group level using the maximum of individual values from the CHC test.

### Outcomes and data collection

#### Primary outcome

The primary outcome parameter is the quality of health information as measured using the MAPPinfo Checklist. Assessment of the primary outcome requires the providers to deliver one work sample in the form of health information. Other information produced during the study period will not be considered. The work sample has to inform a decision in healthcare (e.g. therapy, diagnostic procedure, screening, prevention or rehabilitation). It addresses patients or medical laypersons and discusses at least two options regarding a specified health problem. Material regarding procedures (e.g. how to perform peritoneal dialysis at home) or the healthcare system, resources giving general advice for health and wellbeing as well as (drug) fact(s) boxes without additional text are not appropriate. Despite these requirements, the providers are free to choose the topic and target group of the information. Therefore, they can further develop already planned information. The providers may use external services (e.g. counselling by experts, graphic design, external performing of pilot tests or external literature searches), but they are responsible for the methods applied and for the content and design of the final information.

The quality of the health information is defined as the extent of adherence to the guideline‘s recommendations. This parameter is operationalised by the MAPPinfo instrument that has recently been developed for this very purpose. MAPPinfo corresponds with the recommendations of the guideline and is the first instrument using exclusively evidence-based quality criteria. By the time it is used in our study, MAPPinfo will have passed all the validation steps. The study protocol for the validation study is in the process of publication. Information on reliability, internal consistency, construct validity, criterion validity and divergent validity will then be available. The instrument comprises 21 to 23 test items (depending on the nature of the addressed decision) in four categories:

- Definitions (comprising target group and aim of the health information)
- Transparency (comprising disclosure of authors, financing, conflicts of interest, level of actuality, sources of information and strategies of information gathering)
- Content (comprising explanations of the medical problem, available options and uncertainty, and information about prevalence, (test quality (if relevant), benefit and harms) and
- Presentation (comprising prevalence, test quality (if relevant), benefits, harms, language, patient stories, graphics and gain-loss framing)

The instrument just focusses on criteria accessible through observation of the health information itself and does not consider quality criteria related to development methods or correctness of the contents, which would require further information sources. Adherence to the criteria checked by MAPPinfo is essential but not sufficient to provide material that improves informed decision-making. Non-adherence with one of the criteria may already challenge the entire information process. Therefore, all the criteria should be met. Nevertheless, the instrument provides percentage scores for each category and for each criterion. In this study, scores based on MAPPinfo will be expressed as a percentage of the fully met criteria. Therefore, the items will be coded as fulfilled (100%), partly fulfilled (50%) or not fulfilled (0%). Scores for information quality will be generated (range 0–100%) as an average over all items. MAPPinfo coding works without previous training. The information will be coded by two independent and blinded raters.

#### Secondary outcomes

Due to the pronounced importance of single aspects of the quality concept, it is planned to use a selection of single criteria as secondary outcomes. For this purpose, single criteria are chosen following strong recommendations based either on empiric effects or on ethical considerations:

- CONTENT 5/PRESENTATION 2: ‘Information about possible benefits is provided in an appropriate manner’
- CONTENT 6/PRESENTATION 3: ‘Information about possible harm is provided in an appropriate manner’
- CONTENT 7/PRESENTATION 4: ‘In the case of diagnostic problems: information about the reliability and safety of the test are provided in an appropriate manner’
- PRESENTATION 5: ‘The health information uses a neutral wording/language throughout’
- DEVELOPMENT: ‘The health information target group has been involved in the development process using appropriate methods’

All these secondary outcomes are binary.

#### Sample size

The sample size was calculated for a t test comparing the IG and the CG regarding guideline compliance to EBHI as measured by MAPPinfo. It is assumed that MAPPinfo is normally distributed with the same standard deviation (*σ*) of 10% in both groups. Pretest data from MAPPinfo showed very low ratings for online-available health information. The quality of this information could be improved with just a little effort. Based on these assumptions and a sample size of 26 provider groups (13 in each study group), a quality improvement of 1.5* *σ* corresponding to a very strong Cohen’s effect size can be detected with 90% power using a two-sided significance level of 5% after excluding at the most 10% dropouts. The power was calculated using the statistical software package SAS version 9.4 (PROC POWER).

#### Procedure

During recruitment, both groups will be informed about the requirement to produce a health information item within 10 months, according to the defined criteria.

**Fig. 3:**
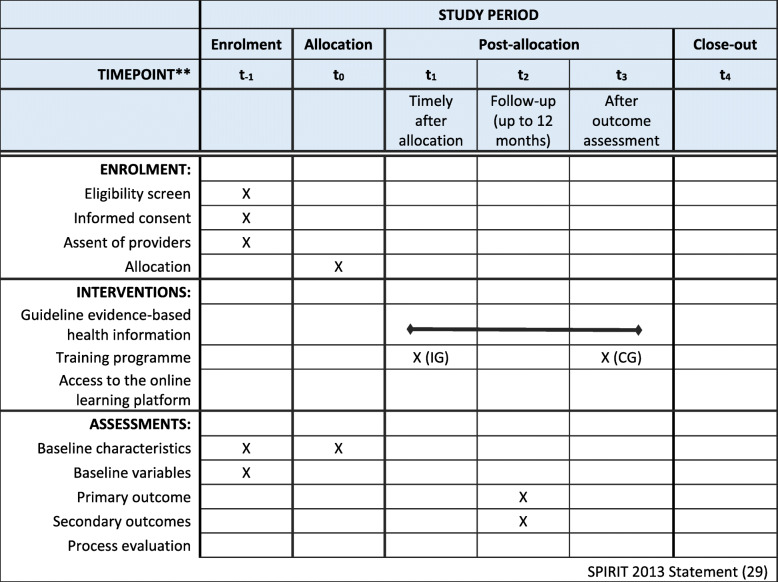
Standard Protocol Items: Recommendations for Interventional Trials (SPIRIT) Figure

In a personal meeting, information on the requirements for study participation will be given and the providers must agree to comply with these requirements. The topic chosen for preparing the work sample will be defined. Written informed consent will be obtained from the individual participants. Consent will be obtained for the assessment of baseline data, for performing a critical health literacy test and for interviews within the process evaluation. Afterwards, baseline data will be assessed (*t*_− 1_) and randomisation will be performed (*t*_0_) (see the SPIRIT Figure, Fig. 3). In the IG, the workshop for the persons responsible (module 0) will be conducted and a timely convenient appointment for the training programme (modules 1 and 2) will be made (*t*_1_). The training should take place on-site at the provider’s institution. If possible in terms of time and place, two or more groups could attend the training together. When the training is completed, they can begin with their production of the work sample. The providers in the CG can start directly after the randomisation. During the time of production, we will contact the providers every 2 months to keep ourselves informed about the progress and to ask about any unexpected difficulties. The study team will be accessible and will answer upcoming questions regarding requirements and the course of the study. The IG will have access to the training material on the online learning platform, including resources such as updated search strategies. We realise that the lack of access to relevant references is an important barrier in the development process and, therefore, the providers in the IG and CG will equally receive support regarding the acquisition of research articles. No further support will be provided.

Completed health information can be sent to the study team at any time. After 10 months, the development process should be concluded and the providers will be asked for the material. A provider who cannot deliver after 10 months can be given a 2-month extension of the deadline. The provider should give a justification for the delay and a reasonable timeline for the following 2 months.

After 12 months (*t*_2_), even unfinished material must be submitted. The information will be rated with regard to the primary outcome. Single criteria (or else the entire information) that do not meet the MAPPinfo requirements, are coded as zero. Providers of both groups (IG and CG) who completed their information will be asked to give further information on the development process (*t*_3_). Interviews will be conducted in the IG to explore barriers and facilitators of implementation (cf. process evaluation). The providers in the CG will be invited to make appointments for the training programme (*t*_3_). After the provided information has been rated, providers may receive individual feedback on their material. This is optional, but this way the feedback would benefit especially the providers in the CG.

#### Data management, protection and analyses

**Fig. 4:**
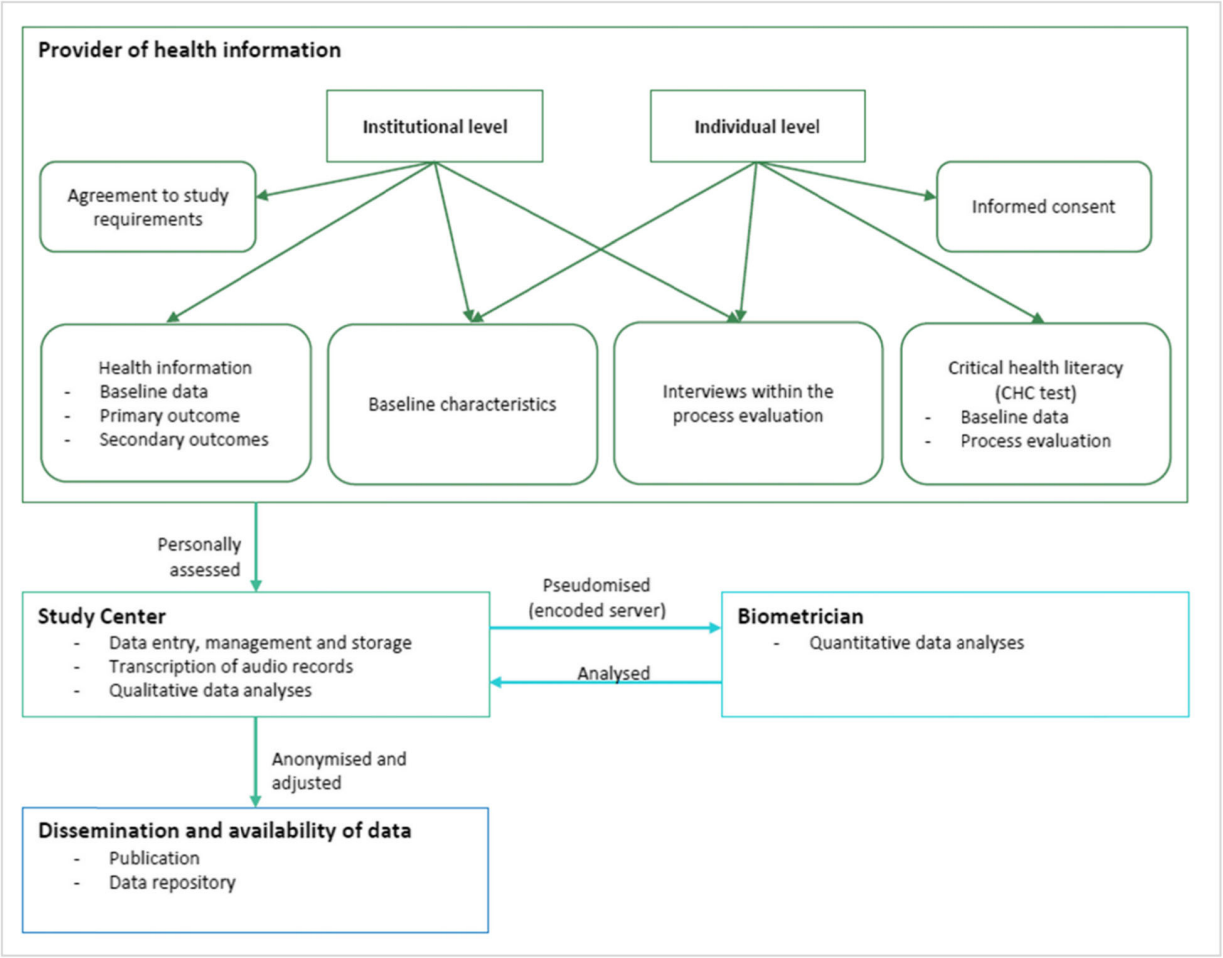
Data flow

Data survey and data processing are used for the scientific purpose of the study only and are appropriate and indispensable for that purpose. Figure 4 shows the data flow. Risk assessment according to §35 General Data Protection Regulation (DS-GVO) revealed a very low risk for the planned study. Special categories containing personal data will not be collected. In addition, the assessment using the Black List/White List of the competent supervisory authority of Saxony-Anhalt did not reveal the necessity for a privacy impact assessment. The remaining very low risk can be further minimised by the planned procedures (e.g. data storage using password-protected servers at the computer centre, no disclosure of data to third parties).

Withdrawal from the study is possible at any time without giving reasons and without disadvantages for the participants. In the case of withdrawal, data can be deleted only before anonymisation (after completion of the analysis). Privacy and data protection will be taken into account according to the German Data Protection Act [37]. A unique code number will be assigned to every participant. All data will be marked with this code number and, thus, pseudonymised. Identification of single participants is only possible with the coding key, which is stored on password-protected servers at the Martin Luther University Halle-Wittenberg. Only the project team has access to the coding key which is protected securely from third parties. Once data analysis has finished, the coding key will be deleted. From then on, data can no longer be deleted in the case of withdrawal from the study.

All data provided by the study participants are treated as absolutely confidential. Researchers adhere to the data protection rules. All data will be stored on password-protected servers and there will be no disclosure to third parties. Publication and storage of data will only be performed in an anonymised form. Audio-recordings will be deleted after transcription. Data will be stored at the Martin Luther University Halle-Wittenberg for 10 years.

The primary analyses will be performed from the intention-to-treat (ITT) perspective. The provider’s characteristics (institutional and individual level) will be analysed descriptively. Baseline variables of individuals will be described on the individual level by means, standard deviations, percentiles and frequency tables, depending on their distributions. The IG and CG will be described separately.

The primary outcome will be analysed on the provider level assuming approximately normal distribution by comparing means between the IG and CG using the *t* est. The distribution of MAPPinfo will be described graphically. In case of deviations from the normal distributions, transformations or alternative tests will be discussed as secondary sensitivity analyses. Frequencies of missing values will be described in detail. Multiple imputations will be performed for the primary outcome if there is enough information available. Only a few missing values are expected because the coding by the raters is very dependable. Incomplete health information will be considered and analysed as non-missing. The imputations will be performed if three values or fewer (12%) of the primary outcome (the outcome score, not the single items) are missing. The multiple imputation model will be fitted using the baseline variables of providers. For the binary secondary outcomes, relative frequencies will be described in both provider groups and compared using Fisher’s exact test. A secondary per-protocol analysis will be performed as a complete case analysis (without multiple imputations for the primary outcome). The secondary per-protocol analysis is planned to compare the results between ITT and per-protocol and to discuss a possible bias from protocol violations and missing values.

A data monitoring committee will not be necessary as the trial does not involve a high-risk intervention and participants do not belong to a vulnerable population. We do not expect adverse events or other unintended effects of the intervention. During the entire study period, participants will be able to contact the study centre, if necessary.

#### Process evaluation

To support future implementation of the *guideline evidence-based health information* and the training programme, a comprehensive analysis of the underlying processes of this complex intervention is indispensable [38]. Barriers and facilitators of implementation should be assessed. Additionally, the high quality of the training programme should be ensured. We will focus on parameters such as recruitment, reasons for participation or non-participation, intervention fidelity, structure and process-related factors, attitudes toward the intervention, response of individuals and organisations, and unintended consequences [38, 39]. Mixed methods will be applied [40] according to the MRC framework for process evaluation of complex interventions [38].

Structured documentation will be used to assess data of recruitment and intervention fidelity (e.g. recruitment process; numbers of institutions invited, responses and participants in each training session; location, time and duration of the training; completeness of modules and online tasks and reasons for deviations; and unexpected difficulties). Feasibility and acceptance of the training programme will be assessed at the end of the training sessions using structured feedback, and all statements will be documented. Critical health literacy will be assessed using the CHC test [36] after the IG and CG have completed the training programme.

To map the development process and the methodological quality of the produced health information, all the providers (IG and CG) will be asked to provide the MAPPinfo self-declaration after completing the health information. The self-declaration comprises free-text questions on the selection of the reported options and outcome measurements, search strategies, methods of data extraction and critical appraisal, reasons for the use of pictures, graphics, fact boxes, value clarification tools, animations and interactive tools. Experts will review the results and, if there are outstanding issues, interviews will be conducted. In addition and to identify influencing factors, the resources and support used in the development process as well as any training attended during the study period will be assessed.

In the IG (theoretical sampling), semi-structured interviews will be conducted with single participants as well as with persons in charge in order to explore the implementation barriers and facilitators. Relevant factors for acceptance and usability of the educational contents and materials may be further assessed. In particular, the use of educational contents in daily routines and barriers against putting the guideline recommendations into practice will be explored. If the participant consents, interviews will be audio-recorded and then transcribed.

Data will be collected at various time points. An iterative process of collecting and analysing qualitative data will allow the exploration of unexpected aspects in further interviews [38]. Data will be analysed in accordance with the method of collection [40]. Descriptive statistics will be used for quantitative data. For qualitative data, a qualitative content analysis according to Mayring will be performed [41].

## Discussion

The aim of the proposed trial is to evaluate the efficacy of the *guideline evidence-based health information* in combination with a training programme for providers of health information in comparison to having access to the guideline on its own. We expect the combined intervention to improve the quality of health information and thus promote informed patient decisions.

However, the training programme is extensive and time consuming; it does not only provide guidance on the application of the guideline but also extensive ebm knowledge. That is because a previous qualitative study revealed the providers’ shortcomings regarding their ebm competences. Participants must be released from their work duties in order to attend the training and, in addition, the development of information in compliance with the guideline recommendations is time and cost-intensive, e.g. comprehensive literature searches must be conducted by the providers themselves or contracted out. The lack of access to databases or references can constitute a further barrier. These potential barriers may limit the study results because of problems in recruitment and study compliance (attending the training programme in person and online; producing information in accordance with the requirements). To address the barriers, the providers will receive comprehensive information on the study course and its requirements, incentives will be provided (publicity for providers of high quality information) and the providers will receive support for purchasing literature. The attitudes of the providers regarding EBHI will play a crucial role and, therefore, these attitudes will be addressed in the training programme and additionally in the workshop for the providers in charge of health information publications. The freedom of choice regarding the topic of the information to be delivered as a working sample might lead to increased internal variance and, therefore, make it more difficult to detect an intervention effect. On the other hand, this method will safeguard both the motivation of the participant to cooperate and the ecological validity. As experience shows the variability on the quality spectrum to be also quite low between topics, we are nevertheless optimistic of being able to demonstrate an effect, even if the small sample size of providers can detect only strong effects of the intervention and limits detailed secondary analyses.

After efficacy is proven, the training programme should be established as a continuous offer for providers of health information in order to enhance their ebm competences and to ensure high-quality EBHI in the long term.

## Dissemination

All the results of the study (including negative ones) will be published in international and open-access journals and presented at meetings and congresses. According to the recommendations of the International Committee of Medical Journal Editors (ICMJE), only persons directly involved in the study will be designated as authors [42].

All the participants will receive an abbreviated version of the final report in language written for laypersons.

## Trial status

Protocol version 2, 26 June 2019.

Date recruitment began: 26 July 2019; approximate date when recruitment will be completed: 31 August 2020.

## Supplementary information

**Additional file 1.** Standard Protocol Items; Recommendations for Interventional Trials (SPIRIT) Checklist.

## Data Availability

The study centre will coordinate the intra-study data sharing process. The data sets will be available for all the principal investigators. After study completion, the adjusted data will be stored and made publicly accessible via a special data repository to fulfil national [43] and international [44, 45] requirements for sharing clinical trial data. For this purpose, the European Open Science Cloud (EOSC), a collaborative tool for data sharing and reuse, was recently implemented to serve all researchers from all research domains [46].

## Abbreviations

CG: Control group
DNEbM: *Deutsches Netzwerk Evidenzbasierte Medizin*
EBHI: Evidence-based health information
ebm: Evidence-based medicine
GMC: General Medical Council
GPGI: *Good practice guidelines for health information*
GRADE: Grading of Recommendations Assessment, Development and Evaluation
IG: Intervention group
MAPPinfo: Mapping Health Information Quality
MRC: Medical Research Council
RCT: Randomised controlled trial
SPIRIT: Standard Protocol Items: Recommendations for Interventional Trials
